# Prevalence of Avian Pathogenic *Escherichia coli* (APEC) Clone Harboring *sfa* Gene in Brazil

**DOI:** 10.1100/2012/437342

**Published:** 2012-04-30

**Authors:** Terezinha Knöbl, Andrea Micke Moreno, Renata Paixão, Tânia Aparecida Tardelli Gomes, Mônica Aparecida Midolli Vieira, Domingos da Silva Leite, Jesus E. Blanco, Antônio José Piantino Ferreira

**Affiliations:** ^1^Faculdade de Medicina Veterinária e Zootecnia, Universidade de São Paulo. Avenue Prof. Dr. Orlando Marques de Paiva, 87 05508-900 São Paulo, SP, Brazil; ^2^Departamento de Microbiologia, Imunologia e Parasitologia, Escola Paulista de Medicina, Universidade Federal de São Paulo, 04023-062 São Paulo, SP, Brazil; ^3^Instituto de Biologia, Universidade Estadual de Campinas (UNICAMP), 13083-970, Campinas, SP, Brazil; ^4^Laboratório de Referência de E. coli (LREC), Departamento de Microbioloxía e Parasitoloxía, Facultade de Veterinaria, Universidade de Santiago de Compostela (USC), 27002 Lugo, Spain

## Abstract

*Escherichia coli sfa*+ strains isolated from poultry were serotyped and characterized by polymerase chain reaction (PCR) and amplified fragment length polymorphism (AFLP). Isolates collected from 12 Brazilian poultry farms mostly belonged to serogroup O6, followed by serogroups O2, O8, O21, O46, O78, O88, O106, O111, and O143. Virulence genes associated were: *iuc* 90%, *fim* 86% *neuS* 60%, *hly* 34%, *tsh* 28%, *crl/csg* 26%, *iss* 26%, *pap* 18%, and 14% *cnf*. Strains from the same farm presented more than one genotypic pattern belonging to different profiles in AFLP. AFLP showed a clonal relation between *Escherichia coli sfa*+ serogroup O6. The virulence genes found in these strains reveal some similarity with extraintestinal *E. coli* (ExPEC), thus alerting for potential zoonotic risk.

## 1. Introduction

 Avian pathogenic *Escherichia coli* (APEC) is considered an outstanding pathogen for the poultry industry, due to several economic losses associated with chronic respiratory disease, septicemia, salpingitis, omphalitis, and embrionary death. Serogroups O2 and O78 are preferentially associated with colibacillosis outbreaks in poultry and represent 80% of disease cases worldwide [1]. These serogroups are well studied. However, little has been researched on the other APEC serogroups with minor importance in the poultry health context, which could, nonetheless, have some impact on public health.

Recently, some authors have suggested the involvement of several mammals and birds species as reservoirs for human extraintestinal pathogenic *Escherichia coli *(ExPEC) serogroups [2–5]. ExPEC strains were characterized as* E. coli* isolates containing two or more of the following virulence markers:* pap*A (P fimbriae structural subunit) and/or* pap*C (P fimbriae assembly),* sfa/foc *(S and F1C fimbriae subunits),* afa/dra *(Dr-antigen-binding adhesion),* kps*MT II (group 2 capsular polysaccharide units), and* iut *(aerobactin receptor) [6]. Many of these virulence factors, found in ExPEC strains that cause human neonatal meningitis (NMEC) and urinary tract infection (UPEC), are also present in APEC, leading to zoonotic concern [7, 8].

S fimbriae encoded by *sfa* operon is a common virulence factor among APEC, NMEC, and ExPEC strains. *E. coli sfa+* strains are very important, since S fimbriae has been related to the pathogenesis of urinary infections, meningitis, and septicemia in human patients. It is estimated that 30 to 60% of human isolates bear gene coding for S fimbriae [9]. The presence of *E. coli sfa+* strains in animals seems to be a rare event, with few reports found in the literature [7, 10–13].

*Escherichia coli sfa+* animal infection was first described by Harel et al. (1991) in F165 *pap+* isolates from calves and piglets with diarrhea and septicemia [10]. In 1992, Dozois et al. isolated two *E. coli sfa+ *strains in avian colisepticemia, and in 1997 the authors isolated fifteen *E. coli cnf+ sfa+* in pigs with diarrhea and septicemia [11, 12]. An epidemiological study involving 1601 *E. coli* isolated from chicken, ducks, and turkeys in Spain, France, and Belgium revealed the presence of 4.2% of positive strains to S fimbriae [7].

 In a previous study, we identified 6% (12/200) of *E. coli* isolates positive carrying S fimbriae, in the colony hybridization test in Brazil [14]. The aim of this study was to characterize *sfa *gene positive *E. coli* strains isolated from Brazilian poultry farms, identifying serogroups, virulence factors, and genotypic profiles, through amplified fragment length polymorphism with a single enzyme.

## 2. Materials and Methods

### 2.1. Bacterial Strains

 A total of 50 strains previously tested by colony blot to the *sfa* gene were selected from avian presenting omphalitis, salpingitis, chronic respiratory disease, and swollen head syndrome, in 10 poultry farms located in five Brazilian states, from 1994 to 2001. All bacterial strains were stored in Lúria Bertani broth with 20% glycerol at −80°C.

### 2.2. Colony Hybridization to the *sfa* and *fim* Genes

The test strains were examined by colony blot hybridization, using specific DNA probes labeled with *α* ^32^ P-d ATP by nick translation [15]. The DNA probe used to detect *fim* B-H genes was a 9.6 Kb *Hin*dIII-*Sal*I fragment from recombinant plasmid pIB254 [16]. *sfa* genes detection involved amplicons obtained by polymerase chain reaction (Table 1) [17]. Positive and negative controls were included in all hybridization assays.

### 2.3. Determining Serogroups

Determination of O and H antigens was carried out with the method described by Guinée et al. (1981), employing all O available (O1 to O181) [18]. All antisera were obtained and absorbed with the corresponding cross-reacting antigens, to remove nonspecific agglutinins. O antisera were produced at Laboratorio de Referencia de *E. coli*, Departamento de Microbioloxía e Parasitoloxía, Facultade de Veterinaria, Universidade de Santiago de Compostela (Lugo, Spain, http://www.lugo.usc.es/ecoli/).

### 2.4. Detection of Virulence Factors by Polymerase Chain Reaction

The polymerase chain reaction (PCR) procedure was applied after DNA extraction, according to the protocol described by Boom et al. (1990) [19]. Reaction mixtures (50 *μ*L) contained PCR buffer (1X), MgCl_2 _(1.5 Mm), 200 mM of each deoxyribonucleotide (dATP, dCTP, dGTP, dTTP), 50 pmol of each oligonucleotide, 1.0 U of Taq DNA polymerase, autoclaved ultrapure water, and 5 *μ*L of DNA template. Primers for specific amplification of the *iuc*, *neuS*, *hly*, *tsh*, *crl/csg*,* iss*, *pap,* and *cnf* genes, annealing temperatures, fragment size, and references are described in Table 1 [20–23]. The amplified products were separated by electrophoresis in 2% agarose gel and stained with ethidium bromide. The 100 bp DNA ladder was used as a molecular size marker.

### 2.5. Single-Enzyme Amplified Fragment Length Polymorphism (AFLP)

 Restriction endonuclease digestion and ligation was performed using a modified method described by McLauchiln et al. (2000) [24]. To 10 *μ*L of the DNA extracted, 24 U of *Hind* III (Invitrogen, Inc.) were added along with ultrapure water, to a final volume of 20 *μ*L, and this reaction was incubated overnight at 37°C. An aliquot of digested DNA (5 *μ*L) was added to 0.2 *μ*g of each adapter ADH1 and ADH2 oligonucleotides, 1 U of T4 DNA ligase (Invitrogen, Inc) along with ultrapure water, to a final volume of 20 *μ*L, and this reaction was incubated at room temperature for 3 hours. Ligated DNA was heated to 80°C for 10 min, and 5 *μ*L were used for each PCR reaction. PCR reactions were performed in 50 *μ*L of the final volume, containing 5 *μ*L of ligated DNA, 2.5 mM of MgCl_2_, 30 pmol of primer (either HI-A, HI-G or HI-T), and 1 U of Taq polymerase, in a 1 X PCR buffer. This mixture was subjected to an initial denaturing step at 94°C for 4 min, followed by 35 cycles of 1 min at 94°C, 1 min at 60°C, and 2.5 min at 72°C. The base sequences of adapter and selective primers were: ADH1—5′ACGGTATGCGACAG 3′, ADH2—5′AGCTCTGTCGCATACCGTGAG 3′, HI-A—5′GGTATGCGACAGAGCTTA 3′, HI-G—5′GGTATGCGACAGAGCTTG 3′, and HI-T—5′GGTATGCGACAGAGCTTT 3′. Electrophoresis was conducted on 1.5% agarose gel at 22 V for 24 hours. The amplified products were visualized with ethidium bromide staining and then were compared to a 100 bp DNA ladder (Invitrogen, Inc).

### 2.6. Statistical Analysis

 The levels of relatedness of the isolates were determined by comprehensive pairwise comparison of restriction fragment sizes, using Dice coefficient. Mean values obtained from Dice coefficients were employed in UPGMA, using the NTSYS pc 2.0 version software to generate the dendrogram.

## 3. Results

*Escherichia coli sfa*+ were isolated from twelve poultry farms located in five Brazilian states.  Table 2 shows the phenotypical and genotypical (PCR and colony hybridization) results (serogrouping).

11 serogroups were identified: O2, O6, O8, O21, O25, O46, O78, O88, O106, O111, and O143. Serogroup O6 was the most frequent, representing 62% of the total number of strains. Serogroups O2, O21, and O78, commonly found in poultry affected by colibacillosis, comprised only 10% of the *sfa+* strains. Three strains could not be identified with the serum tested.

Colony hybridization with *fim operon *revealed 86% of positive strains. Results from PCR revealed that 45 (90%) strains were positive to the aerobactin gene—*iuc*D; 30 (60%) to the K1 gene—*neu*S; 17 (34%) to haemolysin—*hly*A; 14 (28%) to temperature-sensitive hemagglutinin—*tsh*; 13 (26%) to serum resistance—*iss*; 9 (18%) to P fimbriae—*pap*EF, 7 (14%) to the cytotoxic necrotizing factor—*cnf*1. Forty-seven isolates (94%) presented the gene that regulates the curli operon, but only 13 strains (26%) possessed the structural *csg*A gene. None of the strains were positive to nonfimbrial adhesion—*afa*BC. According to the data presented in Table 2, 28 different genetic patterns were observed, with greater concentration of strains among patterns G13 and G12.

**Figure 1:**
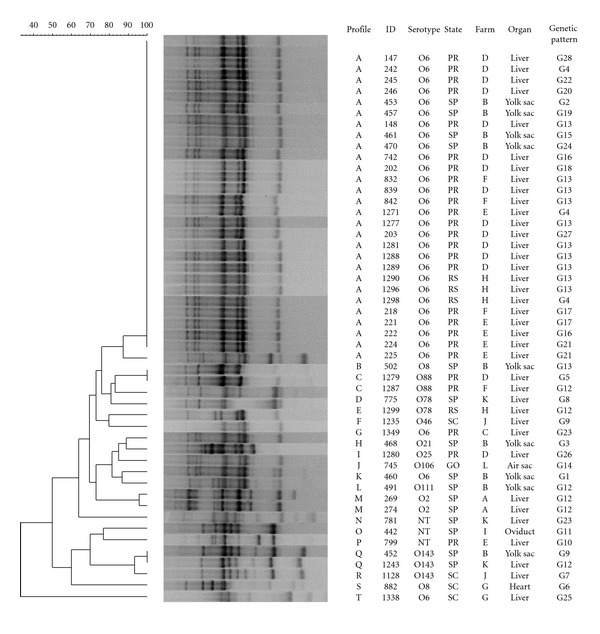
Unweighted pair-group method with arithmetic clustering (UPGMA) dendrogram based on data from AFLP analysis of  *Escherichia coli sfa+. *

Analysis of the samples with AFLP revealed the presence of 20 profiles. Each profile produced from 4 to 10 fragments (bands), with approximately 600 to 3200 bp (Figure 1). Thirty-four (68%) strains generated seven fragments. AFLP produced a dendogram (Figure 1), in which two groups may be identified (Ia and Ib), with lower than 40% similarity rate. Group Ia showed 49 samples distributed in seven subgroups, while group Ib presented only one strain belonging to serogroup O6, classified as T profile. Six subgroups derived from group Ia (IIa; III a, IVa, Va, and VIa) showed 50 to 82% similarity, which resulted in 17 profiles (labeled with the letters C to S), composed by 20 strains. Despite the profile diversity in this dendogram region, one may observe that isolates belonging to the same serogroup were allocated to the same subgroup.

The upper dendogram region showed greater homogeneity, and it is composed by 29 strains classified in profiles A and B, which are derived from subgroup VIIa with higher than 82% similarity. Twenty-eight strains belonging to serogroup O6, and only one from O8, were classified as profile A.

In profile A, 10 out of 11 strains belonging to genotype G13 were grouped, besides 3 strains belonging to G4, and 2 belonging to genotypes G16, G17, and G21. Strains with identical genotypes G9, G12, and G23 patterns, however, were placed in distant dendogram regions.

## 4. Discussion

 In the present study, APEC *sfa+* were characterized phenotypically and genotypically. Eleven serogroups were identified (Table 2). The most common serogroups isolated from poultry (O2, O8, O21, O78, and O88) represented only 16% of the strains analyzed, while the uncommon serogroups (O6, O25, O46, O106, O111, and O143) represented 78% of the total. Many of these are considered human pathogens associated with extraintestinal infections [25].

Table 2 shows that 31 strains (62%) belonged to serogroup O6. Serogroup O6 was a UPEC prototype. Da Silveira et al. (2002) identified 2.3% of APEC serogroup O6 among isolates from chicks with omphalitis [25]. Vandemaele et al. (2003) investigated 100 APEC strains from 83 Belgian poultry farms, detecting only three serogroup O6 strains, all of them positive to the *pap* gene. In this study, the authors related the predominance of *pap*GII allele to different APEC serotypes, alerting for the zoonotic potential of the strains, which are very similar to human isolates [8].

 The occurrence of avian extraintestinal *E. coli* strains positive to the *sfa *gene was previously reported in other parts of world, as described by Stordeur et al. (2002), which detected 4.2% of *E. coli sfa+,* after analyzing 1601 isolates from European poultry farms [7]. Here, *sfa *gene-positive strains were identified at 12 poultry farms, localized in five Brazilian states: SP, PR, SC, RS, and GO. Paraná state presented the highest percentile of positive strains (52%). These results differ from data obtained by Delicato et al. (2003), who report none positive strains for* sfa*DE and *sfa*S, in a study with 200 strains from different farms located in Paraná state [26].

 Many epidemiological studies on avian colibacillosis were based on the comparative frequency of virulence genes, in fecal strains and organs obtained from diseased poultry. In this study, we compared the frequency of virulence genes between traditional APEC strains (reported in the literature) and those positive to the *sfa *gene. The results showed that the frequency of some virulence genes reported here was different from those previously reported [26–28]. The *tsh *gene was detected in 85.3% of the APEC analyzed by Janben et al. (2001), in 39.5% of the strains analyzed by Delicato et al. (2003), and in 53.3% of the strains analyzed by Ewers et al. (2004). In this study, the *tsh+* strains frequency remained at 28%. Similarly, Ewers et al. (2004) detected 82.7% of strains positive to the *iss* gene, Delicato et al. (2003) reported 38.5%, and this study detected only 26%.

 The *pap *gene also was observed at a lower frequency (18%), when compared to the data obtained by Janben et. al. (2001), who found 30% of APEC *pap+.* However, some researchers, such as Delicato et al. (2003), obtained a lower percentage, 18.5% in Brazil, Ewers et al. (2004), 22.7% in Germany, thus suggesting geographical variation for *pap+* strains prevalence [26–28].

 Although *tsh*, *iss, *and *pap* genes have been detected at a lower percentage in the literature, the detection of other genes goes beyond the estimated prevalence. Thirty (60%) strains positive to the gene that encoded for the K1 capsule were identified, while the literature reports a percentage ranging from 8 to 20% for APEC [26].

 Seven strains (14%) were positive to the *cnf*1+ gene. Although genes encoding the presence of cytotoxic necrotizing factors in avian strains have been investigated by various authors, the occurrence of positive strains has not yet been notified [26, 29, 30]. Seventeen strains (14%) were *hly*+, and the occurrence of *hly+* APEC was also uncommon [30–32]. There are no previous studies on the participation of the CNF toxin and hemolysin in poultry colibacillosis pathogenesis. However, the importance of these toxins is very well established for diseases affecting mammals [12, 29]. Data from this study, supported by the APEC literature [1], suggest that the *hly *and *cnf* genes act as a virulence marker of *E.coli* associated to mammals, and their presence in birds should be monitored as an indicator of interspecies barrier transposition.

 According to the data exhibited in Table 2, it was possible to observe 28 genetic patterns, according to virulence gene detection. G13 profile was represented by serogroups O6 and O8, positive to the *sfa, crl*, *iuc, fim,* and *neu*S genes, comprising 22% of the isolates. This profile was identified in four poultry farms in São Paulo, Paraná, and Rio Grande do Sul states. Other frequent profile was the G12 profile, representing 12% of the total number of strains, with the *crl+iuc+fim+neuS+tsh+csg*A*+iss+ *gene combination, and serogroups O2, O78, O88, O111, O143 detected in 3 farms in São Paulo state and 1 farm in Paraná. According to Table 2, isolates from different farms or states could be grouped within the same genetic pattern, and distinct genetic pattern strains were found in the same poultry farm.

 A study involving the genomic subtraction in the APEC serogroup O2, aiming at the identification of new virulence genes, revealed high homology among six of the nine genomic sequences identified with the genes described in human strains, associated with meningitis and urinary tract infection. These observations suggest a clonal relation between avian and human *E. coli* [33].

 Clonal analysis of *Escherichia coli* has been widely employed in epidemiological surveys. Many molecular approaches have been indicated for the discrimination of *E. coli* from avian origin: profiles for plasmid and isoenzymes, ribotyping, restriction endonuclease analysis (REA), pulsed field gel electroforesis (PFGE), random fragment length polymorphism (RFLP), and ERIC PCR [34].

 AFLP has been employed for differentiation of important bacterial species, because this technique can identify disperse mutations in genomic, with the same sensitivity of RAPD or PFGE [35]. AFLP with a single enzyme applied on these 50 strains, grouped them in 20 profiles (Figure 1) with a discriminatory index of 0.68. Geornaras et al. (2001) employed conventional AFLP in a study with 50 *E. coli* isolates from broilers carcass, obtaining a higher discriminatory index. However, they observed an excessive division, with 41 distinct patterns and lower similarity, thus suggesting the absence of clonal relation between carcass and fecal strains [36].

 Dendogram analysis (Figure 1) shows that most of the strains (56%) were located in profile A, characterized by the concentration of serogroup O6. The other 22 strains were grouped in 19 profiles, with 100% of maximum (C-Q profile), and 38% of minimum (T profile) similarity.

 Ewers et al. (2004) employed PFGE for the analysis of 150 APEC strains and reported great difficulty in associating the serogroup and the profiles obtained with virulence genes distribution [28]. Similarly, in this study, it was not possible to establish a correlation between AFLP profiles, genetic patterns, organs of isolation, and strain origin. However, the association between the AFLP profile and serogroup was noteworthy.

 To the A profile, 90.3% of serogroup O6 strains were allocated. The C profile grouped two strains of serogroup O88. Serogroup O78 was allocated to D and E profiles, with 80% similarity, while two O2 isolates with the same origin and genotypic pattern were grouped in M profiles, with 96% similarity. Serogroup O143 strains were allocated to the Q and R profiles, with 94% similarity.

 There is a consensus in the literature as to the existence of limited APEC clones [25, 34, 37]. However, this study evidences a high level of heterogeneity among these strains. In conclusion, the data presented suggest the zoonotic potential of avian *E*.* coli sfa+* APEC. The existence of these strains in Brazilian poultry farms must be investigated by epidemiological surveys, with particular attention to the geographical distribution of serogroup O6 in recent isolates (2002 to 2011).

## Figures and Tables

**Table 1:** The primers used for detection of the various genes by PCR, amplicon size, and references.

Gene	Oligonucleotide primer pairs (5′—3′)	Amplicon (bp)	Reference
*pap*EF	GCAACAGCAACGCTGGTTGCATCAT AGAGAGAGCCACTCTTATACGGACA	336	[20]
*sfa*	CTCCGGAGAACTGGGTGCATCTTAC CGGAGGAGTAATTACAAACCTGGCA	410	[20]
*hly*A	AACAAGGATAAGCACTGTTCTGGCT ACCATATAAGCGGTCATTCCCGTCA	1177	[20]
*iuc*D	TACCGGATTGTCATATGCAGACCGT AATATCTTCCTCCAGTCCGGAGAAG	602	[20]
*cnf*1	AAGATGGAGTTTCCTATGCAGGAG CATTCAGAGTCCTGCCCTCATTATT	498	[20]
*crl*	TTTCGATTGTCTGGCTGTATG CTTCAGATTCAGCGTCGTC	250	[21]
*csg*A	ACTCTGACTTGACTATTACC AGATGCAGTCTGGTCAAC	200	[21]
*tsh*	GGGAAATGACCTGAATGCTGG CCGCTCATCAGTCAGTACCAC	420	[21]
*iss*	GTGGCGAAAACTAGTAAAACAGC CGCCTCGGGGTGGATAA	760	[22]
*neu*S *(kps) *	TATAATTAGTAACCTGGGGC GGCGCTATTGAATAAGACTG	927	[23]

**Table 2:** Serogroup, virulence genes, and genetic patterns of APEC *sfa+. *

Genetic patterns	Virulence genes	Strains (*n*)	Sorogroups	States	Farms
G1	*crl+iuc+fim+neu+hly+tsh+csg*A*+iss+cnf*1*+ *	1	O6	SP	B
G2	*crl+iuc+fim+neu+hly+ iss+cnf*1*+ *	1	O6	SP	B
G3	*crl+iuc+fim+neu+hly+ cnf*1*+ *	1	O21	SP	B
G4	*crl+iuc+fim+neu+hly+*	3	O6	PR/RS	D-E/H
G5	*crl+iuc+fim+neu+csg*A*+iss+pap+ *	1	O88	PR	D
G6	*crl+iuc+fim+neu+csg*A*+iss+pap+ *	1	O8	SC	G
G7	*crl+iuc+fim+neu+tsh+csg*A*+ *	1	O143	SC	J
G8	*crl+iuc+fim+neu+tsh+pap+*	1	O78	SP	K
G9	*crl+iuc+fim+neu+tsh+iss+*	2	O46/O143	SP/SC	B/J
G10	*crl+iuc+fim+neu+csg*A*+ *	1	NT	PR	E
G11	*crl+iuc+fim+neu+tsh+*	1	NT	SP	I
G12	*crl+iuc+fim+neu+tsh+csg*A*+iss+ *	6	O2/O78/O88/O111/O143	SP/PR	A-B-K/F
G13	*crl+iuc+fim+neu+*	11	O6/O8	PR/RS/SP	D-F/H/B
G14	*crl+iuc+fim+hly+csg*A*+cnf*1*+ *	1	O106	GO	L
G15	*crl+iuc+fim+hly+cnf*1*+ *	1	O6	SP	B
G16	*crl+iuc+fim+*	2	O6	PR	E-D
G17	*crl+iuc+fim+hly+*	2	O6	PR	F-E
G18	*crl+iuc+fim+hly+pap+*	1	O6	PR	D
G19	*crl+iuc+hly+iss+cnf*1*+ *	1	O6	SP	B
G20	*crl+iuc+hly+pap+*	1	O6	PR	D
G21	*crl+iuc+hly+*	2	O6	PR	E
G22	*crl+iuc+*	1	O6	PR	D
G23	*crl+fim+neu+*	2	O6/NT	SP/PR	K/C
G24	*crl+fim+hly+tsh+cnf*1*+ *	1	O6	SP	B
G25	*iuc+fim+neu+*	1	O6	SC	G
G26	*fim+neu+tsh+*	1	O25	PR	D
G27	*iuc+hly+*	1	O6	PR	D
G28	*crl+*	1	O6	PR	D
